# UPR Signal Activation by Luminal Sensor Domains

**DOI:** 10.3390/ijms14036454

**Published:** 2013-03-21

**Authors:** Marta Carrara, Filippo Prischi, Maruf M. U. Ali

**Affiliations:** Department of Life Sciences, Centre for Structural Biology, Imperial College London, London SW7 2AZ, UK; E-Mails: marta.carrara08@imperial.ac.uk (M.C.); f.prischi@imperial.ac.uk (F.P.)

**Keywords:** unfolded protein response, ER-stress, signaling

## Abstract

The unfolded protein response (UPR) is a cell-signaling system that detects the accumulation of unfolded protein within the endoplasmic reticulum (ER) and initiates a number of cellular responses to restore ER homeostasis. The presence of unfolded protein is detected by the ER-luminal sensor domains of the three UPR-transducer proteins IRE1, PERK, and ATF6, which then propagate the signal to the cytosol. In this review, we discuss the various mechanisms of action that have been proposed on how the sensor domains detect the presence of unfolded protein to activate downstream UPR signaling.

## 1. Introduction

Eukaryotic secretory and membrane proteins attain their functional and assembled conformation in the endoplasmic reticulum (ER) prior to transit to the Golgi apparatus. A number of molecular chaperones, folding enzymes and co-factors assist in the folding, post-translational modification, and trafficking of the newly translated polypeptide chains. Protein quality control mechanisms, such as the calnexin/calreticulin chaperone system, ensure only properly folded proteins leave the ER [1]. If proteins fail to reach their functional folded state, they are retained in the ER lumen where they are directed towards degradation via the ER-associated degradation (ERAD) pathway, or through autophagy [1,2]. Conditions which perturb the physiological state of the ER, or ER-stresses, can compromise the processing of mature proteins leading to the accumulation of misfolded proteins in the ER lumen and initiation of the unfolded protein response (UPR) [3]. These include nutrient and energy deprivation, hypoxia, alterations in cell redox status, depletion of ER calcium stores, elevated protein trafficking though the ER, altered post-translational modifications, and pathogen infection [3].

**Figure 1:**
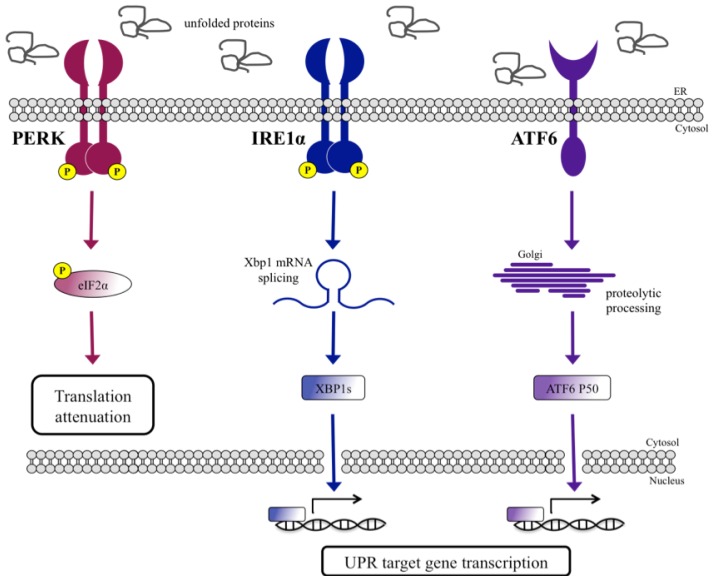
The unfolded protein response in mammals. During ER-stress, the accumulation of unfolded proteins in the ER lumen activates PERK, IRE1 and ATF6. Activated PERK phosphorylates eIF2α resulting in the reduction of protein translation. The active cytosolic domain of IRE1α cleaves and removes an intron from Xbp1 mRNA which then encodes for XBP1s protein. Once activated, ATF6 translocates to the Golgi where it is proteolytically cleaved releasing the active ATF6 P50 fragment. XBP1s and ATF6 P50 transcription factors migrate to the nucleus and activate transcription of UPR target genes.

The UPR was first characterized in *Saccharomyces cerevisiae* where the response to ER stress was found to be mediated uniquely by the inositol requiring enzyme 1 (IRE1) protein (referred to as yIRE1 in this review) [4]. In mammals, UPR signaling is mediated by three ER-localized signal transducers: IRE1 (α and β homologs), double-stranded RNA activated protein kinase (PKR)-like ER kinase (PERK), and activating transcription factor 6 (ATF6) [4]. Each of these proteins has an ER-luminal domain that senses conditions of ER stress, an ER-transmembrane domain, and a cytosolic domain that transmits signals to transcriptional and translational machineries (see Figure 1) The UPR activates cellular mechanisms that attempt to restore the folding capacity of the ER by reducing protein translation to limit the influx of newly synthesized proteins in the already stressed ER lumen and by upregulating UPR target genes. To detect the accumulation of unfolded proteins within the ER, it is thought that an ER-resident Hsp70 chaperone termed BiP (Kar2p in yeast) plays a part in detecting the accumulation of unfolded protein within the ER, although its exact role is unclear. Upon activation of the UPR signal, the cytosolic domain of PERK phosphorylates the eukaryotic initiation factor 2α (eIF2α) which results in the reversible and transient attenuation of mRNA translation [5]. This limits protein expression and prevents overload of the stressed ER lumen. Additionally, ER-stress activates IRE1’s cytosolic RNase domain and targets specifically the X-box binding protein 1 (Xbp1) mRNA (Hac1 mRNA in yeast), and the resultant unconventional splicing causes a frameshift which codes for XBP1s (HAC1 in yeast), a potent transcriptional activator of UPR target genes [6]. These genes encode for proteins that are involved in protein folding, maturation, secretion, and degradation, all of which are essential processes for coping with ER-stress [7–9]. IRE1 also degrades additional mRNAs mainly encoding proteins that transverse the secretory pathway, in a process termed the regulated IRE1-dependent decay (RIDD) [10,11]. RIDD may serve as an additional UPR mechanism to limit the translation of proteins preventing overload of the stressed ER. A third arm of the UPR is initiated by ATF6 during ER-stress. ATF6 translocates to the Golgi apparatus where it is acted upon by site-1 protease (S1P) and S2P proteases to release a cytosolic b-Zip transcription factor, ATF6 P50. The transcription factor migrates to the nucleus where it binds the ER-stress response element (ERSE) promoter sequences to initiate expression of UPR genes in a similar fashion to XBP1s [7]. If the cell is unable to restore ER homeostasis and protein folding ability, then apoptotic pathways are initiated leading to cell death [10].

The luminal senor domains of the three UPR transducers detect the accumulation of unfolded protein within the ER [11]. The sensor domains do not share high sequence similarity; however, induction of ER-stress can activate all three signaling pathways suggesting they have a conserved mechanism of sensing unfolded proteins. This mechanism of activation is not yet understood. There have been numerous biochemical and structural studies proposing differing theories to address this central question, with the models varying in their degrees of involvement of BiP in UPR activation. These modes of activation are discussed below.

## 2. BiP-Dependent Models

### 2.1. BiP Acts as a Negative Regulator of UPR Signaling

**Figure 2:**
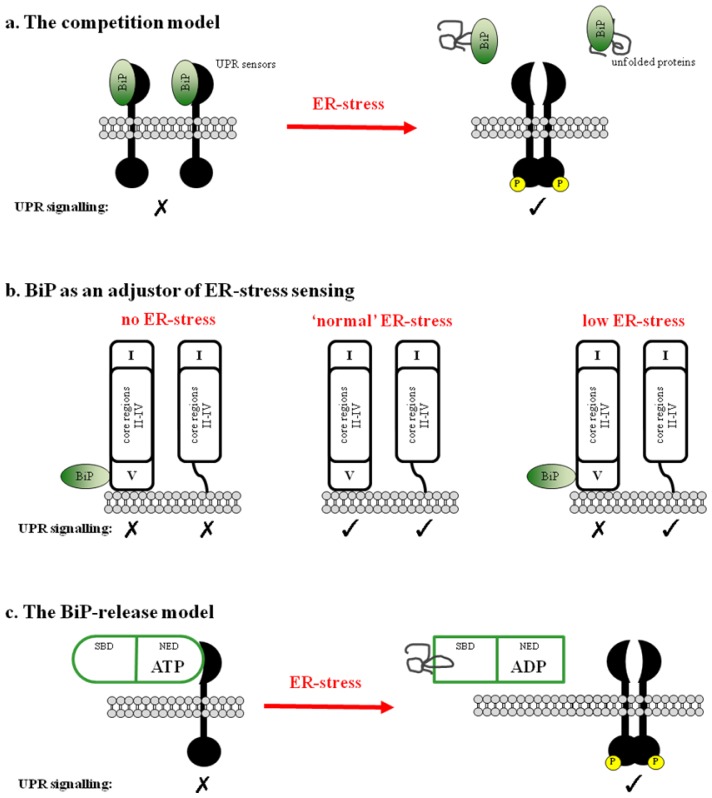
BiP-dependent UPR signaling activation (**a**) The competition model: BiP is normally bound to the ER sensor domains of IRE1, PERK and ATF6 and maintains them in an inactive state. Upon ER-stress, BiP dissociates to bind the unfolded protein substrates and alleviates repression of the UPR sensors allowing for their activation; (**b**) BiP as an adjustor of ER-stress sensing: BiP binds to region V of the ER sensor domains. Loss of BiP binding is not sufficient for activation of UPR signaling in the absence of ER-stress. Rather, “normal” ER-stress sensing requires core regions II and IV, and BiP serves as a fine tuner for instance to deactivate UPR signaling at low levels of ER-stress; (**c**) The BiP-release model: in the absence of ER-stress, ATP-bound BiP binds the luminal sensor domains *via* its nucleotide-exchange domain (NED). During ER-stress, BiP-binding to the unfolded protein substrates *via* its substrate-binding domain (SBD) activates hydrolysis of the bound nucleotide. The resulting conformational changes in BiP cause its dissociation from the sensor domains, which are then free to activate UPR signaling.

The most supported mechanism by which ER-stress initiates the UPR is the “competition model” by which BiP binds to the ER luminal sensor domains of IRE1, PERK and ATF6 in unstressed cells keeping them in an inactive monomeric state. Upon ER-stress, the high concentrations of unfolded proteins compete for BiP binding, thereby sequestering it from the luminal sensor domains. This action allows the sensor domains to dimerize/oligomerize with subsequent propagation of the signal via activation of their cytosolic domains [12,13] (see Figure 2). Correlation between BiP over-expression and attenuation of the UPR was first reported by Droner *et al.* and Kohno *et al.*[14,15]. Early studies in yeast and mammalian transfected cell lines showed that in unstressed cells BiP co-immunoprecipitates with the UPR transducers, and that upon induction of ER-stress, BiP sensor domain complexes dissociate leading to the formation of active IRE1α and PERK oligomers competent for the propagation of the UPR signal [13,16,17]. Furthermore, the over-expression of the ER chaperone calreticulin has no effect on UPR activation, whereas BiP over-expression attenuates UPR signaling [18,19]. Dissociation from BiP also causes ATF6 to translocate to the Golgi, a process required for its cleavage and activation [17]. These data collectively imply a role for BiP as a repressor of IRE1, PERK and ATF6 activation and UPR signaling.

From the studies above, however, it cannot be excluded that senor domain binding to BiP is actually only a client interaction and the decrease in UPR signaling, when BiP is over-expressed, is due to a reduction in levels of unfolded proteins rather than repression of UPR [20,21]. Over-expression of the various constructs may act as an inducer of ER-stress and therefore the observations may not represent physiological conditions of ER-stress. Although this model is the most supported experimentally there seems to be difficulty in differentiating between BiP acting as a chaperone during ER protein folding from its role as an UPR negative regulator. In addition, over-expression of the various constructs used in some studies may itself act as an inducer of ER-stress and therefore the observations may not represent physiological conditions of ER-stress. Indeed, there have been reports that indicate that when expression of IRE1α and PERK is controlled as opposed to over-expressed, full activation of the mutants lacking the BiP binding domain required induction of ER-stress [22,23].

### 2.2. BiP May Act to Fine Tune the UPR Signal

BiP binding was initially thought to occur in the dimerization region of the sensor domains, thereby keeping the senor domain in an inactive state by steric occlusion. BiP release would unmask the dimerization interface allowing dimerization to occur, thus acting as a negative regulator and giving rise to the competition model of activation. This model of repression was also suggested for ATF6, where its ER-stress responsive region contains two Golgi-localization sequences. When bound with BiP, these regions are masked and ATF6 is retained in the ER [17].

The exact model of BiP binding and dissociation from the UPR sensors and how this leads to their activation is not yet understood. In an attempt to identify the BiP binding region, a series of scanning deletion mutants were generated and analyzed for their ability to activate the UPR [20,22,23]. The sensor domains were divided into five regions with regions II and IV, termed the core stress-sensing region, critical for activation of the UPR. Interestingly the BiP binding site was mapped to region V, an area proximal to the ER membrane. Kimata *et al.* show that deletion of region V results in the loss of BiP-binding and that such deletion mutants remarkably have no effect upon UPR signaling and they actually retain their ability to be fully inducible upon ER-stress [23]. In such a scenario, the binding of BiP in region V would only partly contribute to IRE1 and PERK inhibition rather than be the main steric block, thus suggesting a more subtle role for BiP. This was further emphasized by studies which show that a similar BiP deletion mutant (yIRE1 Δ475–526) desensitizes yIRE1’s response to low levels of unfolded protein but has no effect on higher levels of induction of ER-stress [24]. These studies suggest a more peripheral role for BiP in which it possibly acts as an adjustor of ER-stress rather than a role as the direct repressor of UPR signaling (see Figure 2). However, there is a certain degree of ambiguity with some studies indicating that deletion of BiP binding regions actually result in constitutively active UPR signaling in the absence of ER-stress, which would suggest, then, that BiP is indispensable as a negative regulator of UPR signal. This also casts some doubt on region V being exclusively responsible for the UPR sensor domains’ interaction with BiP [20,22,25,26]. Although the phenotype of the BiP deletion mutants would suggest that BiP release is not crucial for activation of the UPR, thus arguing against the BiP release mechanism, the evidence that BiP solely binds region V and that deletion mutants are able to active the UPR is not wholly convincing. It may be that BiP binding requires contributions from other regions of the sensor domains, which would then imply a more direct role for BiP [27].

### 2.3. Regulation of BiP ATPase Activity and the BiP-Release Model

Hsp70 chaperones consist of a nucleotide-exchange domain and a substrate-binding domain, their ATPase activity being key for the control of substrate binding and release. The ATPase activity of BiP may also be important in regulating its binding and release from the luminal sensor domains, thus impacting on UPR activation. In the ATP-bound form, Hsp70s, have an “open” low substrate-affinity conformation, whereas in the ADP-bound form, the substrate-binding domain switches to a “closed” high substrate-affinity conformation, thus trapping the bound substrate. The ATPase activity of Hsp70s is known to be stimulated upon substrate association changing them from an “open” low substrate affinity ATP-bound conformation to a “closed” high substrate affinity ADP-bound form.

Targeted temperature sensitive (ts) mutations present within the ATPase domain lock BiP in “open” ATP-bound state blocking activation of the UPR whilst still associating with IRE1, whereas ts mutations in the substrate-binding domain not only abrogate interactions with unfolded protein substrate but also lead to constitutive activation of the UPR and loss of association with IRE1 [12]. This suggests BiP acts as a negative regulator of the UPR, binding via its substrate-binding domain to the UPR transducers. However, there have been reports that suggest an interaction between nucleotide-exchange domain of BiP with the luminal sensor domains and that this interaction is dependent upon the presence of ATP but not ADP. Mutations that lock BiP in the ATP-bound “open” state were seen to interact with IRE1 consistent with the earlier ts mutations [27,28]. It is known that BiP undergoes large conformation changes within both nucleotide-exchange and substrate-binding domains during its activity cycle. A possible mechanistic scenario is that BiP binds to the sensor domains in the “open” ATP bound state via its nucleotide-exchange domain, leaving the substrate-binding domain free to interact with unfolded protein substrates. Upon unfolded protein binding, ATP is converted to ADP and the corresponding conformational changes would allow the substrate binding domain to adopt the “closed” state and tightly bind the substrate. As a consequence, this would cause the release of BiP from the sensor domain and allow BiP to operate as a chaperone to affect the unfolded substrate [29–31].

In contrast, ATF6 binding to BiP does not seem to depend on nucleotide cycling as their association was not dependent on the addition of specific nucleotides [26]. BiP mutants, which are unable to switch from an ADP to an ATP-bound state, were able to interact with ATF6 in a similar manner to wild type BiP, again suggesting that ATF6 interactions with BiP is not dependent on binding of specific nucleotides [28]. There are differences in BiP binding to ATF6 when compared to IRE1 or PERK, and this is probably due to the low sequence homology in their luminal domains (IRE1 and PERK have a higher degree sequence homology together) and result in subtle differences in mechanisms.

## 3. The Peptide-Binding Model

### 3.1. Direct Binding of Unfolded Proteins to the Luminal Domain of yIRE1

An alternative “BiP-independent” model of UPR activation, which points towards a direct role for unfolded proteins in UPR activation, has been proposed, at least in the yeast system. The 3.0 Å crystal structure of the yIRE1 core luminal domain (residues 114–449) revealed the presence of a groove located at the dimerization interface, interface 1 (IF1), which resembles the peptide-binding domains of major histocompatibility complexes (MHCs) [32]. In its monomeric state, the yIRE1 luminal domain is composed of a triangular assembly of β-sheets that are interspaced by α-helices. Dimerization occurs by symmetric packing of two monomers through polar and hydrophobic interactions between two solvent-exposed antiparallel β sheets creating the MHC-like groove at IF1. Mutation of the putative peptide-binding pocket (M229A-F265A-Y301A) and along IF1 (T226F, F247A) attenuated yIRE1-mediated activation of the downstream target promoter *in vitro*. Interestingly, two further studies have shown direct binding of yIRE1 luminal domain to unfolded proteins and this was a prerequisite for its oligomerization and activation [33,34]. To further characterize peptide binding to yIRE1 luminal domain, Gardner and Walter carried out a peptide array screen derived by sequential titling along the sequence of a constitutively misfolded carboxypeptidase Y mutant (CPY *), which was shown to be a specific yIRE1 substrate [34]. They found that yIRE1 did not recognize a specific consensus sequence, but rather preferentially bound to peptides containing basic and hydrophobic residues that are normally found in the core of folded proteins and become exposed if the peptide chain is not properly folded. They further suggest that peptide binding leads to oligomerization *in vitro* and to activation [34].

### 3.2. Clustering of Active yIRE1 on the ER Membrane

The crystal lattice in the yIRE1 luminal domain crystals revealed the presence of a secondary interaction site: interface 2 (IF2), which may allow for higher oligomer formation in a putative helical arrangement. A mutation at IF2, W426A, also decreased activation of the UPR element (UPRE) reporter, thus suggesting that the clustering of yIRE1 luminal domains via IF2 is involved in the initiation of downstream signaling. Several mutations that compromised yIRE1 cluster formation were shown to disrupt UPR signaling *in vivo*, highlighting the biological relevance of this process at least in the yeast system [33,35]. Gardner and Walter later showed in sedimentation experiments that the binding of unfolded proteins to the MHC-like groove may be the driving force for the clustering of yIRE1 luminal domains [34]. In a mechanistic context, clustering of active yIRE1 may allow cooperativity of its cytosolic domains and activation of downstream signaling pathways [36].

### 3.3. The Peptide-Binding Model in Mammals

Based on the crystal structure of IRE1α luminal domain it was suggested that the direct binding of unfolded proteins is neither probable nor necessary for initiation of the UPR [37]. Zhou *et al.* argue that the MHC-like groove is too narrow and structurally unfavorable to accommodate peptide binding. Firstly, the conserved G105 residue in IRE1α forms a hydrogen bond to contribute to dimer stability, and in doing so, blocks access to the proposed peptide-binding groove. Secondly, two of the residues corresponding to those speculated to contribute to peptide binding in yIRE1, T161 and T179, are either buried or not conserved in the IRE1α groove. Finally, projection of IRE1α cytosolic domain suggests that the MHC-like groove faces the ER membrane thus it is unlikely that protein binding would occur. However, IRE1α was also found to form clusters on the ER membrane of human cells upon induction of ER-stress and this correlated with its phosphorylation and activation of the RNase domain [38]. Another recent study revealed IRE1β to behave more similarly to yIRE1 but not IRE1α in that co-immunoprecipitation with unfolded proteins as well as IRE1β cluster formation were observed in ER-stress activated cells [39]. Based on the crystal structures of IRE1 luminal domains it would appear that divergent mechanisms of sensing ER-stress have evolved in yeast and metazoans. It has been suggested that the two proteins studied may have been trapped in different conformations and therefore represent different states of dimeric IRE1 luminal domains [34]. In addition, both yIRE1 and IRE1α luminal domain crystal structures lack electron density for the ER membrane proximal regions which are speculated to be key for BiP binding making it challenging to draw conclusions on the BiP-dependent model based on the structural information.

## 4. A Two-Step Mechanism for UPR Activation

**Figure 3:**
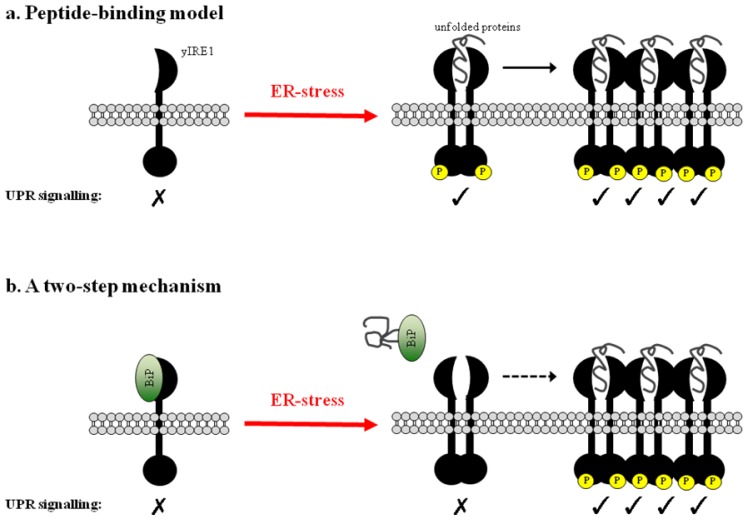
A direct role for unfolded proteins in UPR activation. (**a**) The peptide-binding model: in the absence of ER-stress, monomeric yIRE1 is incompetent for UPR signaling. During ER-stress, the unfolded proteins in the ER lumen bind at the dimerization interface of yIRE1 sensor domain allowing its activation. Peptide binding then leads to the formation of highly active yIRE1 clusters via a secondary interface; (**b**) A two-step mechanism: in this model the dissociation of BiP from the monomeric and inactive yIRE1 sensor domain allows for spontaneous dimer formation. Subsequently, the direct binding of the unfolded proteins at the dimerization interface and yIRE1 clustering activates UPR signaling.

An alternative two-step mechanism has been suggested in which the release of BiP acts as prerequisite to unfolded protein binding to the senor domains [33]. Kimata *et al.* showed that yIRE1 mutants that are unable to form clusters have impaired ability to activate the UPRE reporter, whereas mutants that constitutively cluster still required extrinsic ER-stress to achieve full activation [33]. Together, these results indicate that higher oligomer formation is necessary but not sufficient for UPR activation in yeast cells. Interestingly, deletion mutation of yIRE1 in region I and V (ΔIΔV) were constitutively clustered, whereas ΔI and ΔV single deletion mutants showed normal ER-localization indicating BiP release being necessary for cluster formation [33]. From these observations, it was proposed that BiP senses the presence of and binds to unfolded proteins in the stressed ER lumen, which leads to its dissociation from yIRE1 luminal domain. This induces dimerization, creating the MHC-like groove at IF1 so that the yIRE1 luminal domain is now able to accommodate the unfolded polypeptides [33]. Upon peptide binding, yIRE1 would then undergo a conformational change creating IF2 resulting in the formation of clusters composed of yIRE1 dimers [34,40]. This complex two-step activation mechanism may have evolved as a way of ensuring the tight control of UPR signaling (see Figure 3). However, this mode of activation is quite speculative and would require more experimental evidence to discern the mechanism of action upon peptide binding. Interestingly, the Xbp1 mRNA splicing and RIDD roles of activated IRE1 have been experimentally dissociated [11]. By using different kinase inhibitors to modulate activation of IRE1α’s RNase functions, Han *et al.* were able to uncouple the two downstream signaling branches *in vitro*[11]. It is interesting to speculate that IRE1 at least may have more than one “activated” state, and that different activating signals on the sensor domain, such as BiP dissociation or unfolded protein binding, could influence the nature of downstream signaling events.

## 5. Alternative Mechanisms of Sensing ER-Stress

Recent studies have suggested that IRE1 activation at least can be modulated by extrinsic factors, even those coming from the cytosol. For example, the flavonol quercetin has been shown to dimerize and weakly activate yIRE1 even in the absence of ER-stress. The co-crystal structure revealed quercetin binds to a novel ligand binding pocket of yIRE1’s kinase domain and as such promotes dimerization of yIRE1’s cytosolic domain [41]. Several studies have highlighted the impact of aberrations of the ER membrane, mainly due to imbalanced fatty-acid composition, on UPR activation [42]. Most recently, Kimata *et al.* showed that the cytosolic domain of yIRE1 can sense ER membrane distortions leading to activation of downstream pathways independently of ER-stress [42]. Once again, it is plausible that different ligands and activation signals may have selective effects on the outcome of UPR signaling, such as activation of Xbp1 mRNA splicing or RIDD pathways by IRE1.

Finally, two new and very different mechanisms involved in IRE1α and PERK, and ATF6 activation have recently been proposed. Firstly, Jwa and Chang recently suggested a role of PARP proteins in activation of IRE1α and PERK [43]. PARP proteins can modify acceptor proteins with ADP-ribose modifications and are known to regulate key stress response pathways including DNA damager repair and the cytosolic stress response but, until now, had not been linked to the UPR. In this study, it was found that in the absence of ER-stress, PARP16 was sufficient for activation of IRE1 and PERK. ADP-ribosylation of PARP16, as well as IRE1α and PERK, was shown to increase their RNase and kinase activity, respectively, but did not affect ATF6 activation [43]. Because PARP16 knockdowns impaired the dissociation of BiP from IRE1α and PERK, the authors proposed a mechanism by which PARP16-induced ribosylation could induce conformational changes within the sensor domains, thereby facilitating BiP release and subsequent UPR activation [43].

Furthermore, Hong *et al.* show that glycosylation in the luminal domain of ATF6 serves as a sensor for ER homeostasis and directs ATF6 signaling [44]. The under-glycosylated T654I ATF6 mutant resulted in reduced association with the calreticulin chaperone, faster translocation to the Golgi and increased transcription of target genes [44]. Since glycosylation is greatly compromised during ER-stress and itself is an inducer of the UPR, this study proposes calreticulin association of improperly glycosylated ATF6 as an alternative way of sensing ER-stress and possibly of fine-tuning the UPR response [44]. However it is possible that under-glycosylation actually affected the normal activity of the ATF6 mutant and therefore was responsible for decreased functioning. IRE1 and PERK luminal domains also have *N*-glycosylation sites; however, these have not been linked to ER-stress sensing and their role remains unknown.
